# Impact of Impeller Speed Adjustment Interval on Hemolysis Performance of an Intravascular Micro-Axial Blood Pump

**DOI:** 10.3390/mi15070934

**Published:** 2024-07-22

**Authors:** Yuan Liu, Yuanfei Zhu, Shangting Wang, Hualin Fu, Zhexin Lu, Ming Yang

**Affiliations:** 1 Department of Instrument Science and Engineering, Shanghai Jiao Tong University, Shanghai 200240, China; 2 Department of Cardiovascular Surgery, Shanghai General Hospital, Shanghai 201620, China

**Keywords:** intravascular micro-axial blood pump, hemolysis estimation, finite element simulation, shear stress, flow rate adjustment

## Abstract

Background: In recent years, intravascular micro-axial blood pumps have been increasingly used in the treatment of patients with cardiogenic shock. The flow rate of such blood pumps requires adjustment based on the patient’s physiological condition. Compared to a stable flow state with fixed rotation speed, adjusting the speed of blood pump impeller to alter flow rate may lead to additional hemolysis. This study aimed at elucidating the relationship between adjusting interval of a blood pump’s impeller speed and the hemolysis index. Methods: By comparing simulation results with P-Q characteristic curves of the blood pump measured by experiments, the accuracy of the blood pump flow field simulation model was confirmed. In this study, a drainage tube was employed as the device analogous to an intravascular micro-axial blood pump for achieving similar shear stress levels and residence times. The hemolysis finite element prediction method based on a power-law model was validated through hemolysis testing of porcine blood flow through the drainage tube. The validated models were subsequently utilized to investigate the impact of impeller speed adjusting intervals on hemolysis in the blood pump. Results: Compared to steady flow, the results demonstrate that the hemolysis index increased to 6.3% when changing the blood pump flow rate from 2 L/min to 2.5 L/min by adjusting the impeller speed within 0.072 s. Conclusions: An adjustment time of impeller speed longer than 0.072 s can avoid extra hemolysis when adjusting the intravascular micro-axial blood pump flow rate from 2 L/min to 2.5 L/min.

## 1. Introduction

Cardiogenic shock is a condition caused by severe heart pump failure [1], resulting in acute peripheral circulatory failure [2,3]. Despite rapid advancements in clinical medicine over the last several decades, the short-term mortality rate associated with cardiogenic shock remains around 50% [4]. Percutaneous left ventricular assist devices (PLVADs), such as the intra-aortic balloon pump (Getinge) and Impella (Abiomed Europe, Aachen, Germany), can be rapidly deployed in patients with cardiogenic shock complicating myocardial infarction or acute heart failure [5]. Impella is a type of intravascular micro-axial blood pump. In recent years, Impella has been increasingly used for left ventricular unloading during VA-ECMO, a treatment method known as ECPELLA [6]. However, when compared to the use of VA-ECMO alone, ECPELLA has been observed to increase the incidence of severe bleeding, limb ischemia, and hemolysis [7]. Roberts et al. [8] discovered that incorrect placement of the Impella CP can lead to more severe hemolysis. Therefore, when using the Impella in clinical applications, consideration must be given to the hemolysis caused by [9]. In clinical application, adjusting the speed of the blood pump impeller according to the patient’s physiological status is necessary. During the ECPELLA process, the Impella flow rate initially is set to the maximum flow level. Then, the speed of the Impella is adjusted between P3 (33,000 rpm) and P5 (37,000 rpm) depending on individualized patient factors in an attempt to minimize hemolysis associated with the device while maintaining LV venting capabilities [10]. F. Shu found that the time derivative of flow (dQ/dt) is a very important determinant of the flow field in artificial organs [11]. Impeller speed modulation can lead to higher shear stress levels compared to constant flow conditions, thereby resulting in increased hemolysis [12]. The flow rate of a blood pump undergoes frequent fluctuations during pulsatile operation mode through adjusting impeller speed. Research indicates that continuous-flow left ventricular assist devices may result in gastrointestinal bleeding, arteriovenous malformations, and aortic insufficiency, whereas pulsatile flow left ventricular support has been demonstrated to be advantageous and may be deemed essential for prolonged support [13,14]. The hemolysis index of a blood pump in pulsatile mode is higher than that in constant mode, and the proper adjustment of impeller speed is of crucial significance for reducing hemolysis [12,15]. Elevated hemolysis within the blood pump occurs when the impeller speed increases or the flow rate decreases [16], caused by the increased shear stress and the prolonged residence time of blood in the pump, respectively. Some studies have investigated the impact of the internal flow field of centrifugal blood pumps on hemolysis and platelet activation under pulsatile operation modes [17,18,19]. C. Chen et al. [20] investigated the hemolysis conditions of implantable blood pumps under pulsatile blood flow operation mode. The findings indicated that hemolysis does not exhibit a significant increase when suitable control methods are applied. However, most current studies focus on evaluating the hemolytic performance of centrifugal blood pumps under a constant flow rate. Research related to pulsatile flow rate has mainly focused on the influence of pulsatile modes on hemolysis in blood pumps, and the relationship between the pump hemolysis index and the impeller speed adjusting interval has not been investigated.

To address the abovementioned issues, the research content in this study is mainly divided into three parts: 1. conducting in vitro hemolysis experiments to validate the accuracy of the hemolysis simulation model; 2. testing the performance of the intravascular micro-axial blood pump designed in this research and verifying the accuracy of a flow field simulation model based on experimental results; and 3. investigating the impact of impeller speed adjusting intervals on hemolysis in the blood pump using the simulation model.

## 2. Materials and Methods

### 2.1. Hemolysis Prediction Method Based on Power-Law Model

In this research, blood is simplified as an incompressible Newtonian fluid. Although blood is a viscous non-Newtonian fluid, its viscosity decreases at high shear rates [21]. The interior of a blood pump represents a high shear rate environment, so simplifying blood as a Newtonian fluid is reasonable. Hemolysis under mechanical stress is primarily caused by the rupture of red blood cells under shear stress. Using a fluid–structure interaction method to simulate damage to red blood cells is accurate when predicting hemolysis [22], but this approach requires a significant amount of computational resources. The hemolysis index of blood pumps is commonly calculated using a power-law model. Although the power-law model may not precisely capture the hemolysis values (mainly because hemolysis does not occur when the shear stress is less than a certain threshold value) [22], it can still reveal the variation pattern in hemolysis indices under different operating conditions, and the computational cost is acceptable. Hence, this study continues to utilize the power-law model for computing the hemolysis index. The general form of the power-law model is:

(1) $$D=\frac{∆Hb}{Hb}=A\tau^{b}t^{c}$$

where *D*, *τ*, *Hb*, ∆Hb, and *t* represent the hemolysis index, equivalent shear stress, total concentration of hemoglobin, concentration of free hemoglobin, and exposure time of blood in the shear stress field, respectively. *A*, *b*, and *c* are empirical parameters obtained by fitting experimental data. According to Giersiepen et al. [23], *A* = 3.62 × 10^−7^, *b* = 2.416, *c* = 0.785. The equivalent stress τ is proposed by Bludszuweit et al. [24] based on the von Mises criterion:

(2) $$\tau ={\left[\frac{1}{6}\sum_{\begin{array}{c} i,j=1,2,3\\ i\neq j \end{array}}{\left(\sigma_{ii}-\sigma_{jj}\right)}^{2}+\sum_{\begin{array}{c} i,j=1,2,3\\ i\neq j \end{array}}\sigma_{ij}^{2}\right]}^{\frac{1}{2}}$$

Substitute Equation (2) into Equation (1), then linearize the hemolysis index D in Equation (1) using $D_{I}$ ($D_{I}=D^{\frac{1}{C}}$):

(3) $$D_{I}=A^{\frac{1}{c}}\tau^{\frac{b}{c}}t$$

The power-law model is converted into a convection–diffusion equation according to a material derivative equation:

(4) $$\frac{d}{dt}D_{I}=\left(\frac{\partial }{\partial t}+\vec{v}\cdot \nabla \right)D_{I}=A^{\frac{1}{c}}\tau^{\frac{b}{c}}=I$$

The convection–diffusion equation of the power-law model is:

(5) $$\left(\frac{\partial }{\partial t}+\vec{v}\cdot \nabla \right)D_{I}=I$$

where $\vec{v}$ is the velocity vector, ∇ is the 3D differential operator, and ${I=A}^{\frac{1}{c}}\tau^{\frac{b}{c}}$. This equation was integrated into ANSYS Fluent (2021 R2) for computation using a user-defined scalar (UDS) and was solved by Fluent automatically.

### 2.2. Design and Simulation of Intravascular Micro-Axial Blood Pump

#### 2.2.1. Intravascular Micro-Axial Blood Pump Design and Hydraulic Performance Test

**Figure 1:**
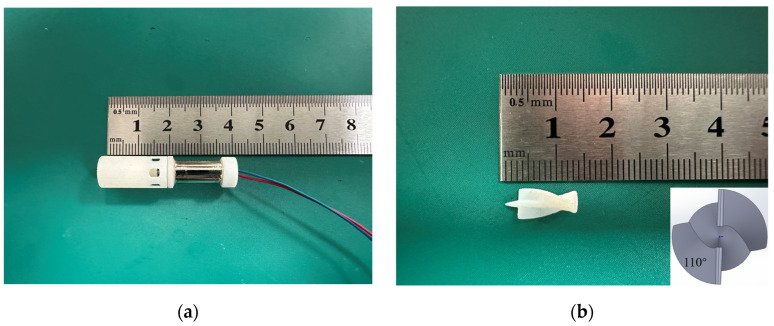
The intravascular micro-axial blood pump used in this study: (**a**) blood pump appearance; (**b**) impeller of blood pump.

We developed an intravascular micro-axial blood pump for conducting in vitro circulation experiments. The blood pump mainly consists of an impeller, a casing, and a driving motor (as shown in Figure 1). The blood pump is manufactured using 3D printing with resin material, featuring a 0.3 mm gap between the blades and the housing. The impeller is comprised of a main shaft and two blades, which are formed by sweeping along a helix with equal cross-section diameter. The angle between the blade tip and tail is 110°. In this study, experimental flow–pressure curves were compared with the flow–pressure curves obtained from finite element simulations to validate the accuracy of the flow field simulation results of the blood pump. The hemolytic characteristics of the blood pump were primarily assessed using simulation results and further validated through hemolysis experiments with a drainage tube with 3 mm diameter. The reason for not directly conducting hemolysis tests using the intravascular micro-axial blood pump was that the blood pump is currently in its early stage of development, and the materials of the pump casing and impeller do not meet the biocompatibility requirements.

**Figure 2:**
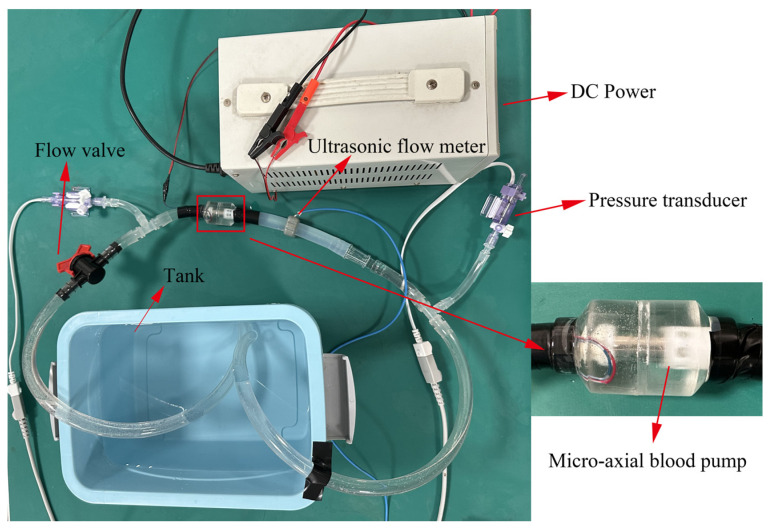
The hydraulic performance test device of the intravascular micro-axial blood pump.

The hydraulic performance test device of the intravascular micro-axial blood pump is shown in Figure 2. We tested the hydraulic performance of the blood pump using pure water, with a density of 1.0 × 10^3^ kg/m^3^ and viscosity of 1.005 × 10^−3^ Pa·s at room temperature. Flow rate was measured by an ultrasonic flow meter. Pressure sensors were installed at the inlet and outlet of the pump to measure the pressure raise generated by the blood pump. The flow control valve modifies the flow rate and pressure within the pipeline while maintaining a constant impeller speed.

#### 2.2.2. Simulation Settings of Intravascular Micro-Axial Blood Pump

**Figure 3:**
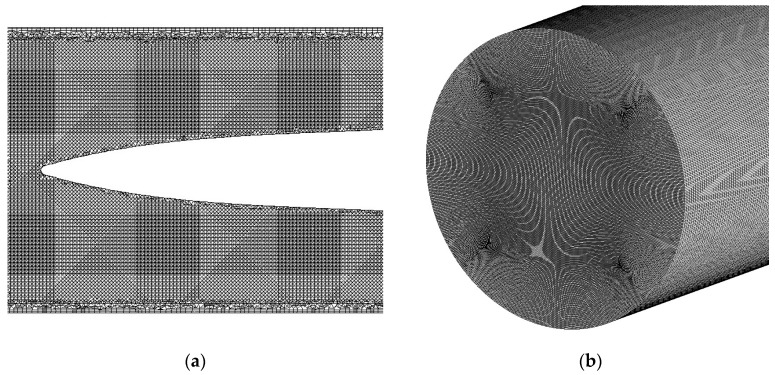
Grid partition scheme: (**a**) mesh of intravascular micro-axial blood pump; (**b**) mesh of drainage tube with 3 mm diameter.

The blood pump was generated by SolidWorks 2021 and meshed using the commercial meshing software ICEM CFD (2021 R2). Due to the complex geometry of the blood pump, tetrahedral unstructured meshes were utilized to discretize the computational domain. To capture flow field intricacies, a prism grid was used near the outer shell wall, with a refined grid utilized in the vicinity of the blades. After the grid was partitioned, grid independence validation was conducted using three different grid resolutions: 5 million, 7 million, and 9 million cells. The discrepancy in outlet flow between the 7 million and 9 million cell grids was found to be less than 2%, and the error of averaged scalar shear stress on the cross section at the midpoint in the longitudinal direction of the blood pump was 2.7%. Considering both computational accuracy and resource utilization, the 9 million-cell grid was selected for computation. The mesh of the blood pump is illustrated in Figure 3a.

In this case, blood density was set to 1050 kg/m^3^, and the viscosity was set to 0.0035 Pa·s [20]. The physical parameters of water were utilized in simulation to validate the hydraulic performance of the blood pump. For simulating the hemolysis index of the blood pump, the physical parameters of blood were used. The blood pump is configured with a pressure inlet and pressure outlet, with the pressure field generated by the rotation of the impeller driving the blood flow. According to hydraulic experimental results, when flow resistance in the hydraulic performance test device is 0, the pressure difference between the inlet and outlet is 10 mmHg. The rotation of the impeller is achieved using a sliding mesh method, with the hemolysis index set to 0 at the inlet. A turbulent model was employed to calculate the flow field and hemolysis within the blood pump, with the Reynolds-averaged Navier–Stokes (RANS) method applied for turbulent model resolution. The k-omega model is an empirical model consisting of two equations based on turbulent kinetic energy and specific dissipation rate utilized for solving the turbulent viscosity in RANS equations with high accuracy near the walls. This study utilized the SST k-omega model for domain resolution. This model combines the standard k-omega model for near-wall flow field resolution and the k-epsilon model for higher-Reynolds-number regions, leading to more precise computational outcomes compared to the standard k-omega model [25]. The simulation model with a transient model was calculated by a workstation using an Intel Xeon Gold 6226R CPU with 32 cores (Intel, Santa Clara, CA, USA), and the computing time step was 1 × 10^−4^ s.

### 2.3. Drainage Tube with 3 mm Diameter: CFD Study Settings

In this study, a drainage tube with a diameter of 3 mm and a length of 80 mm was employed as the device for inducing shear stress. The dimensions were selected to achieve shear stress levels and residence times like those of an intravascular micro-axial blood pump (the inner diameter of the blood pump is 6 mm, and the length is 20 mm) when blood flows through the drainage tube at an equivalent flow rate.

The drainage tube with 3 mm diameter model was partitioned using a structured hexahedral mesh consisting of 9 million cells (shown in Figure 3b). The simulation model was solved using the commercial simulation software Ansys Fluent (2021 R2). In subsequent hemolysis experiments, the flow rate of the blood pump was 1.25 L/min, resulting in turbulent flow conditions within the drainage tube with 3 mm diameter (Re = 3150). Consequently, an SST k-omega turbulent model was employed to calculate the flow field and hemolysis within the drainage tube with 3 mm diameter.

### 2.4. Hemolysis Experiment Settings Based on Drainage Tube with 3 mm Diameter

A total of 500 mL blood was extracted from healthy experimental pigs via femoral artery puncture. The whole-blood cell counts of the blood fell within physiological ranges. Sodium citrate with a concentration of 13.15 g per 500 mL of blood was employed as the anticoagulant for the blood. The interval between blood collection and experimentation did not exceed 12 h, and the blood was stored in a refrigerated environment at 4 °C before experiments. The parameters and storage conditions of the blood are presented in Table 1. Prior to the start of the experiment, the water bath temperature for the blood bag was set to 37 °C. Fill the circulation system and blood pump with PBS and circulate for 10 min to ensure adequate wetting of the tubing. Subsequently, drain the PBS and connect the blood bag containing porcine blood to the circulation system.

**Figure 4:**
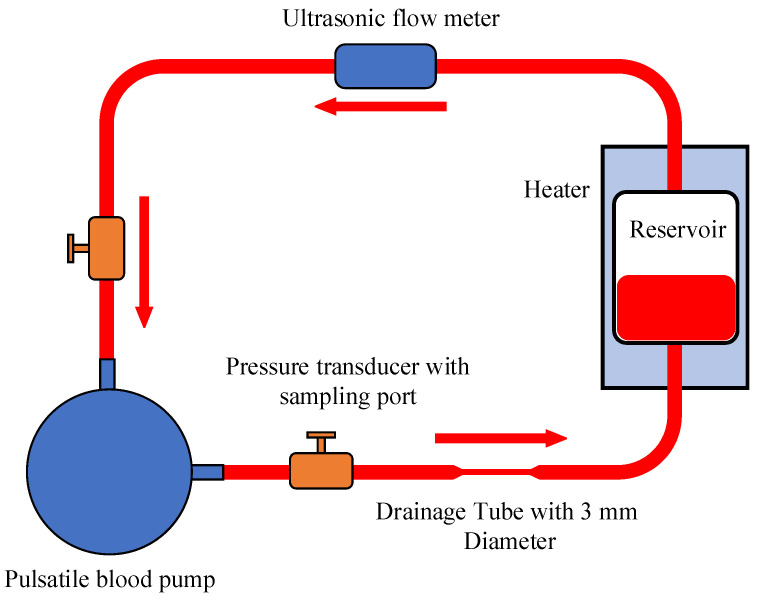
The circulatory system of the hemolysis experiment.

The circulatory system is depicted in Figure 4, red arrows in the figure represent the blood flow direction. To minimize the potential influence of hemolysis induced by the blood pump, a pulsatile blood pump equipped with bioprosthetic valves [26] was employed in this study. Pressure sensors with blood collection ports were positioned at the inlet and outlet of the pump, and blood samples were obtained using single-use syringes from the blood collection ports. Prior to the experiment, the initial level of free hemoglobin in the samples was measured. Subsequently, in accordance with the ASTM F1841-19 standard [27], 5 mL blood samples were collected every 60 min. Each sample was then centrifuged and the free hemoglobin levels were determined using a spectrophotometer. During each sampling, the first 1 mL of blood at the sampling port was discarded to eliminate any potential impact of blood contamination on the experimental results.

## 3. Results

### 3.1. Simulation Result of Drainage Tube with 3 mm Diameter and Blood Pump

**Figure 5:**
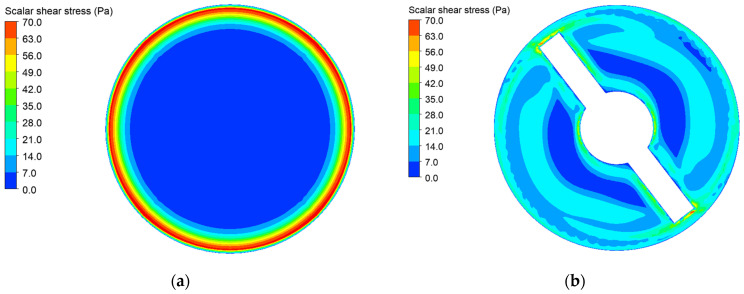
The shear stress distribution on the cross sections at the midpoint in the longitudinal direction of the drainage tube with 3 mm diameter and the blood pump under a blood flow rate of 1.25 L/min: (**a**) shear stress of drainage tube with 3 mm diameter; (**b**) shear stress of intravascular micro-axial blood pump.

The shear stress distribution on the cross sections at the midpoint in the longitudinal direction of the drainage tube with 3 mm diameter and the blood pump under a blood flow rate of 1.25 L/min is illustrated in Figure 5. The mass-weighted averaged hemolysis index at the outlets of the tube and blood pump was $5.79\times 10^{-5}$ and $7.74\times 10^{-5}$, respectively. It can be observed that the hemolysis indices for the drainage tube and intravascular micro-axial blood pump fall within a comparable range. Consequently, employing the drainage tube with 3 mm diameter as an equivalent device for the analysis and simulation of hemolysis in the intravascular micro-axial blood pump is acceptable.

### 3.2. Results of Hemolysis Experiment Based on Drainage Tube

**Figure 6:**
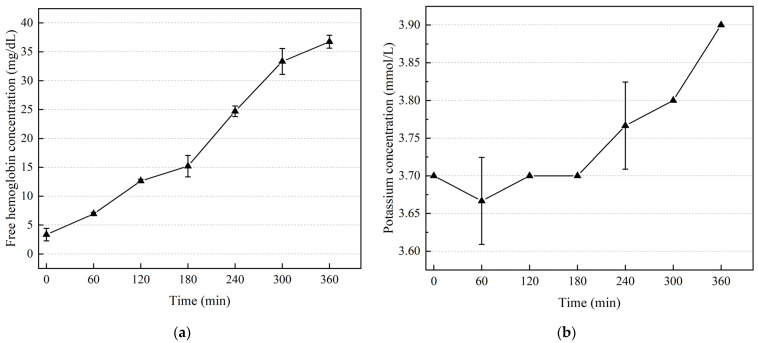
In vitro hemolysis experiment results: (**a**) trend of free hemoglobin concentration in blood over time; (**b**) trend of potassium concentration in blood over time.

To reduce random errors, three blood samples were collected at each time point to measure the free hemoglobin and potassium concentration. The trend of free hemoglobin concentration in blood over time is illustrated in Figure 6a. Beyond 180 min, the increasing rate of free hemoglobin concentration surpasses that observed during the initial 0–180 min interval, indicating more pronounced hemolysis occurring after 180 min. This can be explained as a result of accumulation of red blood cell damage [28]. The potassium concentration in the blood was measured using a blood gas analyzer. Three samples were collected at each measurement point to reduce measurement errors. As shown in Figure 6b, fluctuations in the potassium concentration measurements were observed only at the 60 min and 240 min time points, while the three measurement results of potassium concentration at other times exhibited good consistency, indicating no fluctuation. Figure 6b illustrates a noticeable increase in potassium concentration in the blood circulation after 180 min. This trend is consistent with the rising trend of free hemoglobin. The rising potassium concentration can be attributed to the fact that the concentration of potassium within red blood cells is higher compared to that in the plasma [29]. Upon red blood cell rupture, potassium in red blood cells is released into the plasma, resulting in an elevation in the potassium concentration in blood.

### 3.3. Comparison of Hemolysis Simulation and Experimental Results

*HI* is the hemolysis index in percentage and can be expressed as:

(6) $$HI\left(\%\right)=\frac{∆Hb}{Hb}\times 100$$

where Hb and ∆Hb are the total concentration of hemoglobin and concentration of free hemoglobin, respectively.

**Figure 7:**
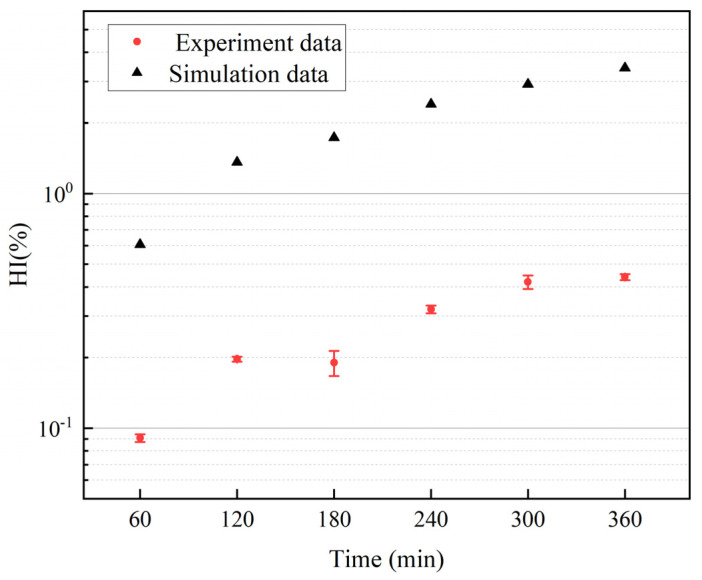
Comparison between the hemolysis index obtained from simulation using a power-law model and the values measured in experiments.

As shown in Figure 7, the hemolysis index obtained from simulation using a power-law model is compared with experimental results. According to the research of Wu et al. [30], the difference between simulation and experimental results in this study falls within an acceptable range. The graph demonstrates that while the simulation results may not precisely predict hemolysis values, they can still reflect the trend of hemolysis changing over time. Consequently, employing the power-law model for simulating the hemolytic performance of the intravascular micro-axial blood pump is acceptable.

The normalized index of hemolysis (*NIH*) serves as a quantitative indicator for assessing hemolytic properties. This parameter represents the increase in grams of plasma free hemoglobin per 100 L of blood pumped, corrected for plasma volume using hematocrit and normalized by flow rate and circulation time. The normalized index of hemolysis derived from simulation can be computed using the following formula [31]:

(7) $${NIH}_{simu}=HbD\left(\tau ,t\right)\times 100$$

where *Hb* is the blood hemoglobin concentration and *D* (*τ*, *t*) is the hemolysis index calculated by simulation.

The ASTM F1841-19 standard [27] specifies the procedures and methods for experimentally measuring and calculating the normalized hemolysis index. The formula for computing the normalized hemolysis index is:

(8) $${NIH}_{exp}=\frac{∆PfH*V*\frac{\left(100-Hct\right)}{100}}{Q*∆T*1000}$$

where ∆PfH, V, Q, Hct, and ∆T are the variation in free hemoglobin concentration in plasma over the sampling time interval, blood volume within the experimental device, flow rate, hematocrit, and sampling time interval, respectively.

Table 2 presents the NIH derived from experimental data. Blood volume within the circuit, flow rate, and time intervals were measured once at each time point, while free hemoglobin was measured three times. Three NIH values were computed for each time instant, and the final NIH was obtained by averaging all NIH values calculated across different time points. The simulated NIH was 0.206 g/100 L, and the experimental value was 0.0248 ± 0.0132 g/100 L. The differential in NIH values between simulation and experimentation can be elucidated by the overestimation of the hemolysis power-law model, as depicted in Figure 7.

### 3.4. Experimental and Simulation Results for Intravascular Micro-Axial Blood Pump

**Figure 8:**
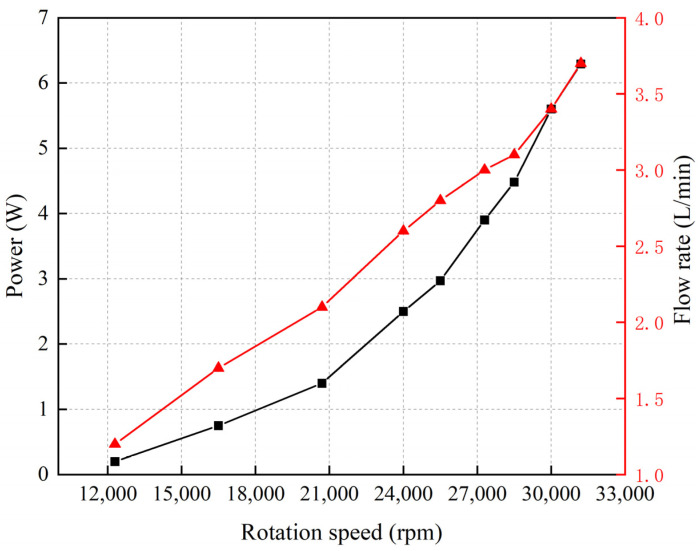
Relationship between rotation speed, power, and flow rate of intravascular micro-axial blood pump.

The motor used in this study has a maximum allowable voltage of 3.7 V, and the motor’s speed can reach 31,200 rpm. Figure 8 illustrates the relationship between the experimental results for speed, power, and flow rate of the intravascular micro-axial blood pump. This experiment was conducted with the valve controlling the flow rate fully open in the hydraulic performance test device. The pressure difference between the inlet and outlet of the pump fluctuated within the range of 10 mmHg to 15 mmHg at various impeller speeds. The graph indicates that as the impeller speed increases, the power and flow rate of the blood pump show an exponential relationship with speed, similar to the findings reported by B. Letzen et al. [32].

**Figure 9:**
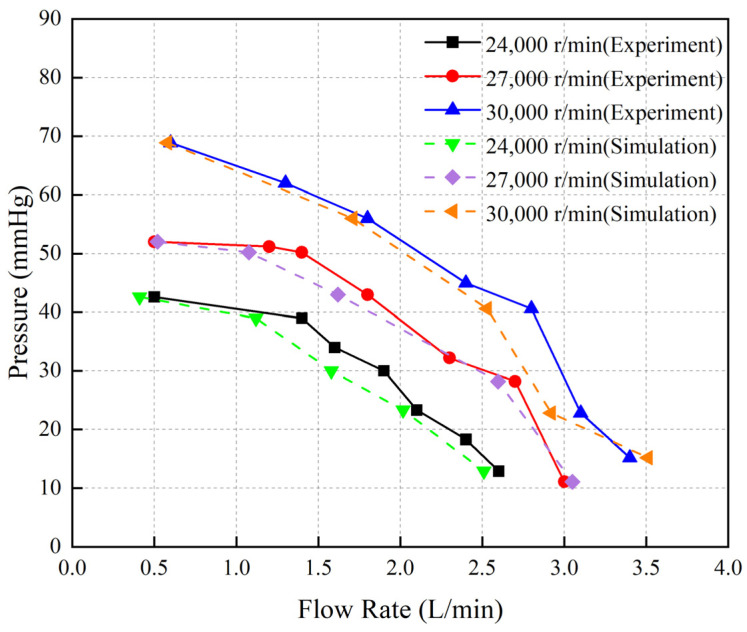
The experimental and simulated results of the P-Q curve for the blood pump.

Figure 9 illustrates the experimental and simulation results of the P-Q curve of the blood pump. The pump’s maximum output flow rates at three different speeds were recorded as 2.5 L/min, 3 L/min, and 3.5 L/min, respectively. The close agreement between the simulation and experimental results demonstrates the capability of the simulation model to accurately reflect the flow field within the pump.

The effectiveness of the hemolysis prediction method was validated by hemolysis experiments using a drainage tube with 3 mm diameter. By comparing the simulated and experimentally measured P-Q curves, we confirmed the accuracy of the simulation model in calculating the flow field of the blood pump. We then employed the power-law model-based simulation model to investigate the influence of the impeller speed adjusting interval on the hemolytic performance of the blood pump.

**Figure 10:**
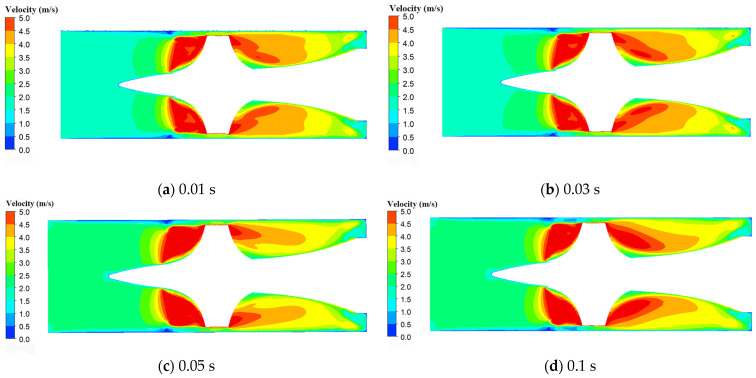
The distribution of the blood flow velocity on the axial cross section within the blood pump in different impeller speed adjusting intervals: (**a**) impeller speed adjusting interval of 0.01 s; (**b**) impeller speed adjusting interval of 0.03 s; (**c**) impeller speed adjusting interval of 0.05 s; (**d**) impeller speed adjusting interval of 0.1 s.

During clinical use of intravascular micro-axial blood pumps, adjustments to the impeller speed are frequently required. In ECPELLA treatment, the rotation speed of the impeller can be adjusted to change the flow rate from 2 L/min to 2.5 L/min based on the physiological state of the body [10]. Based on this typical working condition, we simulated the shear stress within the pump and hemolysis index when the flow rate changes from 2 L/min to 2.5 L/min by adjusting the impeller speed with different intervals. Figure 10 shows the distribution of the blood flow velocity on the axial cross section within the blood pump at different impeller adjusting intervals of 0.01 s, 0.03 s, 0.05 s, and 0.1 s. Figure 10a reveals that at 0.01 s impeller speed adjustment, the blood flow velocity between the impeller and the pump casing gap exceeds that of the other conditions, such as in Figure 10b–d. This gap is the primary location where hemolysis occurs in the blood pump.

**Figure 11:**
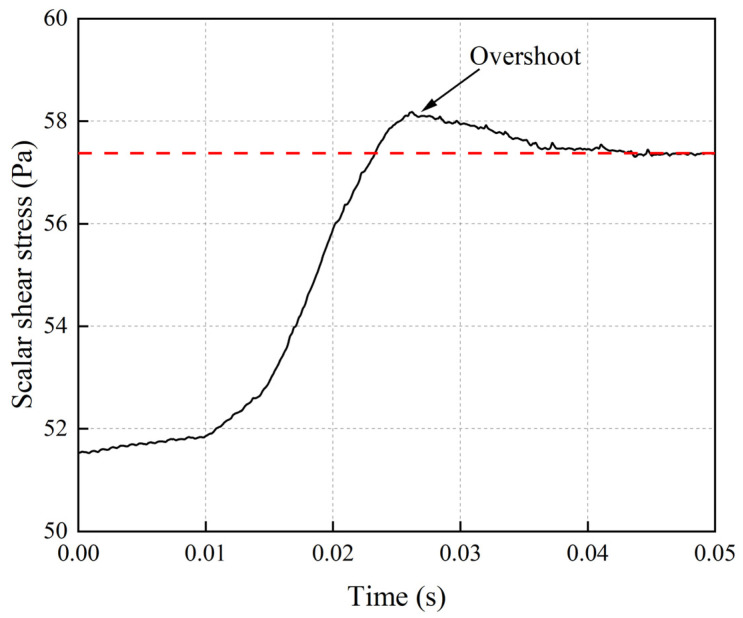
The average scalar shear stress variation on the radial cross section at the midpoint in the longitudinal direction of the blood pump when the flow rate transitions from 2 L/min to 2.5 L/min within 0.01 s. The red dash line is average scalar shear stress on the cross section of blood pump at constant flow rate of 2.5 L/min.

Figure 11 shows the average scalar shear stress variation on the radial cross section within the pump when the flow rate transitions from 2 L/min to 2.5 L/min within 0.01 s. The graph illustrates an overshoot phenomenon in the scalar shear stress, reaching a maximum value of 58.2 Pa before gradually stabilizing. This suggests that rapid impeller speed variations can lead to elevated shear stresses.

**Figure 12:**
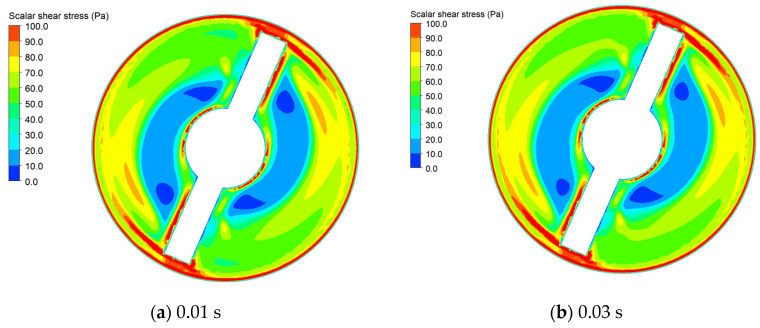

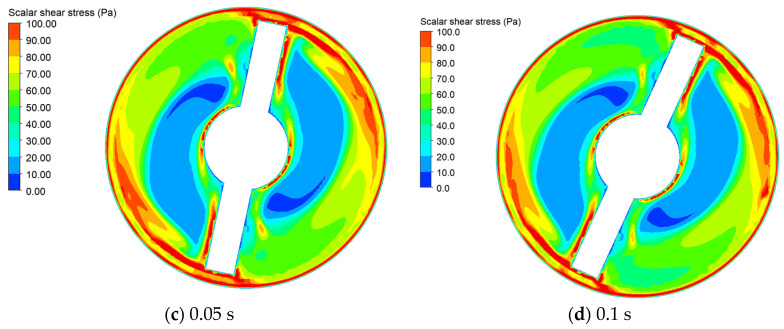
The shear stress distribution on the radial cross section at the midpoint in the longitudinal direction of the blood pump for different impeller speed adjusting intervals: (**a**) impeller speed adjusting interval of 0.01 s; (**b**) impeller speed adjusting interval of 0.03 s; (**c**) impeller speed adjusting interval of 0.05 s; (**d**) impeller speed adjusting interval of 0.1 s.

Figure 12 displays stress distribution on the radial cross section within the blood pump when the average scalar shear stress reaches its maximum values at impeller speed adjusting intervals of 0.01 s, 0.03 s, 0.05 s, and 0.1 s. From the graph, it can be observed that the region with the highest shear stress is located near the wall and blade surface of the blood pump, while stress levels are lower in other areas. This phenomenon is explained by the fact that shear stress is primarily generated by velocity gradients within blood. Positions closer to the wall surface exhibit larger velocity gradients, hence resulting in higher shear stress. The graph reveals that with prolonged impeller speed adjusting intervals, the area with shear stress below 30 Pa near the center gradually increases, while the high-stress regions near the outer walls diminish.

**Figure 13:**
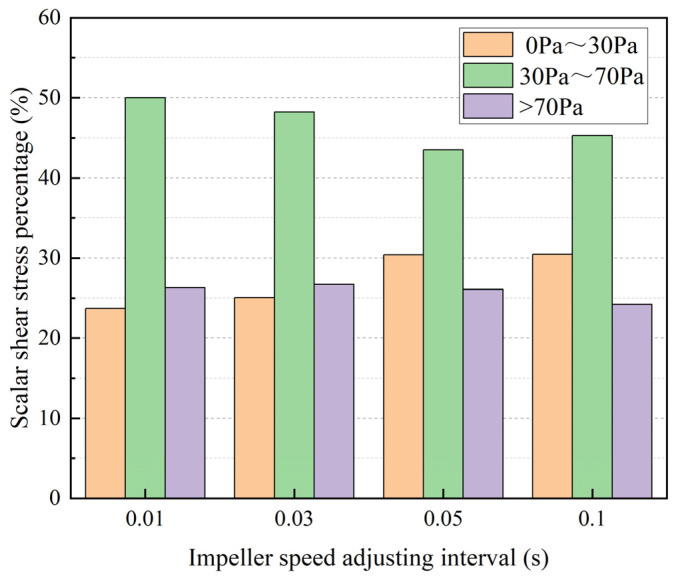
The area proportions of scalar shear stress within various ranges on the radial cross section at the midpoint in the longitudinal direction of the blood pump under different impeller speed adjusting intervals.

We conducted a statistical analysis of the area proportions of scalar shear stress within various ranges on the radial cross section at the midpoint in the longitudinal direction of the blood pump under four different impeller speed adjusting intervals, as illustrated in Figure 13. The shear stress was categorized into three regions: high shear stress (>70 Pa) area, moderate shear stress area (30–70 Pa), and low shear stress (0–30 Pa) area. It is evident from the graph that as the duration of impeller speed adjusting intervals increases, the proportion of the high-shear-stress area on the section decreases gradually, while the proportion of the low-shear-stress area increases progressively. In the hemolysis prediction method based on the power-law model, the hemolysis index is positively correlated with shear stress, as demonstrated in Equation (1). Consequently, as the high-shear-stress region diminishes, the hemolysis index of the blood pump is also expected to decrease.

**Figure 14:**
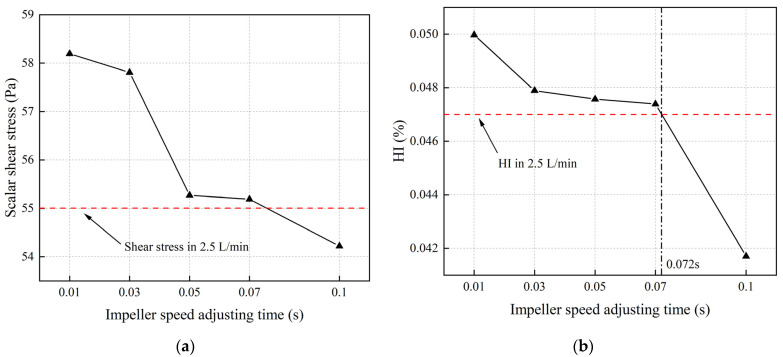
Average scalar shear stress and hemolysis index on the radial cross section at the midpoint in the longitudinal direction of the blood pump during impeller speed variations at different time intervals (The red dashed line representing the average scalar shear stress on the cross section and the hemolysis index at the pump outlet with a flow rate of 2.5 L/min. The black dotted line represents the impeller speed adjusting interval that correspond to the same hemolysis index as at a stable flow rate of 2.5 L/min): (**a**) average scalar shear stress on the radial cross section; (**b**) hemolysis index on the radial cross section.

Figure 14a depicts the average scalar shear stress on the radial cross section at the midpoint in the longitudinal direction of the blood pump during impeller speed variations in different time intervals, with the red dashed line representing the average scalar shear stress on the cross section at a flow rate of 2.5 L/min. The graph indicates that for impeller speed adjusting intervals less than 0.07 s, the time-averaged shear stresses during the impeller speed adjustment process exceed the shear stress value at a constant flow rate of 2.5 L/min. Figure 14b illustrates the hemolysis index at the pump outlet for different impeller speed adjusting intervals. The graph demonstrates a similar trend to the scalar shear stress, showing that when the flow rate variation time is less than 0.072 s, the hemolysis index at the outlet surpasses that at a constant flow rate of 2.5 L/min. Comparing shear stress with the hemolysis index in Figure 14, it can be noted that the shear stress on the cross section starts decreasing when the impeller speed adjustment interval reaches 0.05 s, while the hemolysis index begins to decline when the interval reaches 0.03 s.

## 4. Discussion

We developed an intravascular micro-axial blood pump and assessed its hydraulic performance. Utilizing a validated hemolysis model, we computed the hemolysis index as the pump’s flow rate was adjusted from 2 L/min to 2.5 L/min by adjusting the impeller speed at various time intervals. The results indicate that when the impeller speed adjusting interval is less than 0.072 s, adjusting the impeller speed results in additional hemolysis. This phenomenon arises from the rapid fluctuations of the impeller speed, prompting corresponding changes in fluid velocity within the pump. The drastic variation in flow velocity leads to an elevation in the velocity gradient within the pump’s flow field. It is well known that the velocity gradient within fluids is the primary source of shear stress. Therefore, when the impeller speed changes rapidly, the increased velocity gradient generates higher magnitudes of shear stress, consequently causing greater hemolysis within the pump. This finding is similar to [17], where they found that the artificial pulse increased turbulence substantially and that the resulting elevated total stresses might contribute to hemocompatibility-related problems observed in the clinics.

To our knowledge, there is no research investigating the relationship between the blood pump impeller speed adjusting interval and hemolysis index. Most current research is concentrated on examining hemolysis in blood pumps operating under various constant flow conditions. Some studies have investigated hemolysis and platelet activation of blood pumps under pulsatile operating conditions [17,19,20]. These studies focused on comparing pulsatile flow with steady flow or comparing different pulsatile waveforms, mainly using centrifugal pumps. Our study findings align with the conclusions drawn from these inquiries, suggesting that rapid fluctuations in blood flow velocity caused by adjusting impeller speed in a short interval can contribute to increased hemolysis in an intravascular micro-axial blood pump. The research findings suggest that when regulating the pump flow rate within permissible physiological parameters, increasing the duration of flow rate variations can help mitigate additional hemolysis and reduce the burden on the patient’s kidneys. This observation holds important implications for intravascular micro-axial blood pump manufacturers, highlighting the importance of considering impeller speed adjusting intervals when designing flow rate variation modes.

Our study still has some limitations. This pump is still in its preliminary stages and does not yet meet biocompatibility standards. Consequently, our research utilized a simulation model to predict the hemolysis index. Our study focused only on conditions where the blood flow changed from 2 L/min to 2.5 L/min. Additional research is required to explore whether more significant changes in flow rate (e.g., from 2 L/min to 3 L/min) necessitate longer impeller speed adjustment intervals to avoid extra hemolysis.

## 5. Conclusions

The findings of this study suggest that for equipment manufacturers, the blood pump’s impeller speed adjustment time interval must be considered according to the hemolysis index. In designing the pulsatile mode of the blood pump, restrictions should be placed on the increasing time of the pulsatile waveform to prevent unnecessary hemolysis. Further investigation will be focused on the relationship between impeller speed adjusting intervals and blood pump hemolysis index across various flow rate ranges with a refined intravascular micro-axial blood pump.

## Figures and Tables

**Table 1:** The parameters and storage conditions of porcine blood.

Item	Parameter
Retention time	12 h
Anticoagulant	Sodium citrate
Hematocrit	36%
Temperature	4 °C

**Table 2:** Normalized index of hemolysis (NIH) calculated from experimental data.

Time (min)	NIH (g/100 L)	NIH (g/100 L)	NIH (g/100 L)	Average (g/100 L)
60	0.016	0.0178	0.0156	0.01647
120	0.0289	0.0267	0.0297	0.02843
180	0.0055	0.0069	0.0196	0.01067
240	0.0461	0.0401	0.0385	0.04157
300	0.0266	0.0454	0.042	0.038
360	0.0085	0.0149	0.0169	0.01343

## Data Availability

The original contributions presented in the study are included in the article. Further inquiries can be directed to the corresponding author.
